# Opioid Prescribing Practices and Training Needs of Québec Family Physicians for Chronic Noncancer Pain

**DOI:** 10.1155/2017/1365910

**Published:** 2017-07-31

**Authors:** Élise Roy, Richard J. Côté, Denis Hamel, Pierre-André Dubé, Éric Langlois, Maud Emmanuelle Labesse, Christiane Thibault, Aline Boulanger

**Affiliations:** ^1^Faculté de Médecine et des Sciences de la Santé, Université de Sherbrooke, Campus de Longueuil, 150 place Charles-Le Moyne, Room 200, Longueuil, QC, Canada J4K 0A8; ^2^Direction des Risques Biologiques et de la Santé au Travail, Institut National de Santé Publique du Québec, 190 Crémazie Est., Montréal, QC, Canada H2P 1E2; ^3^Bureau d'Information et d'Études en Santé des Populations, Institut National de Santé Publique du Québec, 945 avenue Wolfe, Québec City, QC, Canada G1B5 5V3; ^4^Direction de la Santé Environnementale et de la Toxicologie, Institut National de Santé Publique du Québec, 945 avenue Wolfe, Québec City, QC, Canada G1B5 5V3; ^5^Direction de la Santé Environnementale et de la Toxicologie, Institut National de Santé Publique du Québec, 190 Crémazie Est., Montréal, QC, Canada H2P 1E2; ^6^Clinique Antidouleur du Centre Hospitalier de l'Université de Montréal, 1560 rue Sherbrooke Est., Montréal, QC, Canada H2L 4M1

## Abstract

**Aim:**

To examine medical practices and training needs of Québec family physicians with respect to pain management and opioid prescription for chronic noncancer pain (CNCP).

**Methodology:**

An online survey was carried out in 2016.

**Results:**

Of 636 respondents (43.0% men; 54.3% ≥ 50 years old), 15.2% and 70.9% felt very or somewhat confident that they could properly prescribe opioids for CNCP. Concerns related to abuse (72.5% strongly/somewhat agree), dependence (73.2%), and lack of support (75.4%) were the main barriers reported. Only 19.7% always/often screened their patients for risks of abuse and dependence using a screening tool. About two-thirds of participants (65.7%) had recently (last five years) taken part in continuing education programs on opioid use for CNCP and 73.4% on CNCP management. Patient evaluation and differential diagnoses of chronic pain syndromes were rated as a top priority for further training.

**Conclusions:**

This study provides insights into Québec family physicians' concerns, practices, and needs with respect to the management of CNCP. Physicians' difficulties around the application of strategies to mitigate the problem of opioid abuse and addiction are worrying. The need to better train physicians in the field of pain and addiction cannot be emphasized enough.

## 1. Introduction

Chronic pain is a significant health problem in many countries with prevalence rates ranging from 10% to more than 50% [1–3]. One area of major concern is the high related societal costs, estimated to be in billions annually in many western countries [4, 5]. In Canada, the most recent estimate of the prevalence of chronic pain among adults was 18.9% in 2007-2008 [3]. Healthcare costs (excluding societal costs related to disability and lost productivity) can be expected to rise from $6.02 billion to $10.29 billion per year by 2025 [6].

Few studies have assessed the long-term benefits of opioids for chronic pain [7]. Yet, opioids are commonly prescribed and a sharp increase in prescriptions has been observed over the last two decades in North America [8, 9]. This has resulted in major adverse public health consequences ranging from diversion, abuse, dependence, and addiction to fatal overdoses [10–14]. In the province of Québec, where this study took place, a report from the Institut National de Santé Publique du Québec revealed that, between 2000 and 2012, mortality due to poisoning by opioids increased by 6.5% a year in men and 8.7% a year in women [13, 15]. In Canada, recent work by Fischer et al. (2014) showed that there was considerable heterogeneity among provinces regarding quantities and types of prescription opioids (POs) dispensed and that Québec was the province with the lowest dispensing level [16]. The study did not allow explaining these differences and, apart from Canadian studies that included small numbers of Québec physicians [17, 18], little is known about Québec physicians' beliefs and practices with respect to chronic noncancer pain (CNCP) management. The most recent study, carried out in 2009, looked at a small sample (*n* = 137) of physicians identified as analgesics prescribers and assessed their knowledge, attitudes, and beliefs regarding CNCP [19]. Results showed suboptimal scores on all aspects of pain management, including initial assessment, definition of treatment goals and expectations, development of a treatment plan, and reassessment and management of longitudinal care. In 2009, the Collège des Médecins du Québec posted guidelines for opioid use for chronic pain [20]. Since then, no studies have examined Québec physicians' current practices and training needs in the field.

In light of this knowledge gap, this study sought to determine whether physician prescribing habits conform to Collège des Médecins du Québec guidelines for opioid prescription for chronic noncancer pain, to identify barriers to opioid use in the treatment of chronic noncancer pain, and to assess training needs of physicians regarding pain management and prescription of pain medication. This study falls under the mandate of l'Institut National de Santé Publique du Québec and its partners, the Collège des Médecins du Québec (CMQ), the Centre de Recherche et d'Aide pour Narcomanes, and the University of Sherbrooke; this study was funded by Health Canada in 2014.

## 2. Methods

An online survey was carried out among physicians practicing in the province of Québec in 2015. Participants who identified themselves as family physicians and reported that they prescribed opioids for chronic noncancer pain were selected for this study. The Montréal Public Health Department's Research Ethics Board provided a waiver from ethics review for the survey.

### 2.1. Questionnaire Design

The questionnaire design was based on a review of the literature including scientific papers and, when available, study questionnaires that addressed topics relevant to the survey objectives. A list of over a hundred questions was developed; an initial selection was made, based on the study objectives and the context of Québec physicians' practices (e.g., type of practice setting). A set of questions—used as originally formulated or adapted—was assembled into a first draft of the study questionnaire (in French) and then submitted for consultation to a group of experts in the field including an anesthesiologist who heads a pain clinic, a family doctor trained in pain management, and a pharmacist. The group was asked to check the length of the questionnaire, which was not to exceed 15 minutes, its fluidity, clarity, and potential for bias. A new version was produced and then reviewed by Ipsos, an independent market research company hired to support the research team. The final version of the questionnaire was translated into English by Ipsos, and the translation was verified by two physicians whose mother tongue was English.

The questionnaire was divided into sections pertaining to each of the study objectives. Section 1 included three questions on certification in pain medicine, methadone, and buprenorphine prescribing. An additional question addressed the number of patients the physician treats per week and the percentage of these patients requiring CNCP intervention. Section 2 collected information about perceived barriers to prescribing as well as physicians' practices and compliance with the CMQ guidelines. Potential barriers were examined by assessing physicians' levels of concern about a series of factors that might cause a physician to avoid or become hesitant about prescribing opioid analgesics for CNCP. Physicians were also asked if they felt confident that they could properly (1) prescribe opioid analgesics for CNCP and (2) treat patients with CNCP. For both series of questions, they indicated whether they strongly agreed, somewhat agreed, somewhat disagreed, or strongly disagreed with the statement.

Two questions addressed physicians' choices of medication in cases of mild to moderate pain. Several options of opioids were proposed in their short- or long-acting versions, including codeine with or without acetaminophen, hydrocodone, hydromorphone, meperidine, morphine, oxycodone with or without acetaminophen, tramadol with or without acetaminophen, butorphanol nasal spray, tapentadol, methadone, buprenorphine transdermal patch, and fentanyl transdermal patch. The following products were proposed as possible adjuvants for coanalgesia: acetaminophen, anticonvulsants, antidepressants, nonsteroidal anti-inflammatory drugs, hypnotic sedatives or tranquilizers, medical marijuana, medical synthetic cannabinoid, and muscle relaxants. Participants were asked how often they prescribed any of those medications as the first-line treatment, using a four-item Likert scale with the following choices: always, often, occasionally, and never. No precision was provided to participants regarding the frequencies to which these choices corresponded.

Using the same four-item Likert scale, compliance to CMQ guidelines was assessed, asking participants how often they did each of a series of 12 actions, listed in the CMQ guidelines. Section 3 asked about clinicians' appreciation of the quality of their academic training in the field of pain and whether they had participated in related continuing education programs in the past five years. Physicians were also asked to identify their perceived learning needs and preferences regarding the format and content for a continuing education program. To do so, they were asked to rank the main topics out of eight by training priority and to do the same with a list of special populations (e.g., adolescents, pregnant women, and CNCP patients with mental health disorders) for whom they might want to receive training in CNCP management. Lastly, participants were asked to rank different tools in terms of relevance to facilitate decision-making as to whether or not opioid analgesics should be prescribed. Questions in Section 4 addressed the physicians' sociodemographic profile (age, sex, and practice location) and practice characteristics (specialty, practice type, and patient volume).

### 2.2. Data Collection

Physicians were invited to participate in the survey through a “newsletter” that the CMQ sent by e-mail to all its members; it described the survey and included information about anonymity. According to the CMQ, 20,983 physicians out of a total of 22,153 subscribed to the newsletter. Physicians were invited to click on a link that brought them to an intranet web site managed by Ipsos. Only physicians who practice clinical medicine were invited to fill out the whole questionnaire.

To improve participation, the CMQ sent the invitation twice, one week apart. The survey was also promoted through several channels including the Fédération des Médecins Omnipraticiens du Québec's newsletter, personal solicitation of the presidents of several medical specialty associations of the Fédération des Médecins Spécialistes du Québec, and the professional services of the Centres Intégrés de Santé et de Services Sociaux and Centres Intégrés Universitaires de Santé et de Services Sociaux, thus including all health and social services at the core of Québec's public health system.

### 2.3. Statistical Analyses

Data were analyzed using weighted proportions according to age and sex distribution of all Québec family physicians. For the Likert scales, proportions are reported for single or aggregated categories. Analyses were performed using SAS 9.4.

## 3. Results

The survey took place between October 14, 2015, and November 17, 2015. A total of 1,111 individuals completed the questionnaire, but 19 were excluded because of incomplete or incoherent answers. Of the 1,092 participating physicians, 653 identified themselves as family physicians, including 636 who reported that they prescribed opioid analgesics at least occasionally to their patients with CNCP. Given the convenience sampling strategy used in this survey and the anonymity of the process, it is not possible to estimate the rate of refusal to participate. According to the CMQ database, 9,285 family physicians were active during the survey period; therefore, the participation rate is estimated to be 7.03%. Whether remote or urban, all health regions were well represented, with almost a quarter of the sample (24.56%) practicing on the island of Montréal.  Table 1 shows that the sex and age distribution of the overall family physician population and the sample were similar.

Table 2 describes the clinical practice profile of survey participants. The majority (74.9%) had been practicing for 10 years or more at the time of survey and most were quite active with over two-thirds (66.9%) reporting that they treated at least 50 patients a week. A significant number (43.7%) were practicing in hospitals, half in family medicine groups, about a third in private clinics, and a quarter in community clinics. More than half of the participants were involved in clinical teaching (54.8%) and nearly 20% had an exemption to prescribe methadone, mostly for pain. To the question on number of patients seen in a week, 33.0% of the physicians answered 49 and under, 50.6% answered 50 to 99, 14.8% answered 100 to 149, and 1.6% answered 150 and over. For each of these categories, the proportions of patients requiring interventions for CNCP were estimated to be 85.9%, 87.1%, 89.5%, and 62.7%, respectively.

The majority of participants felt at ease working in the field of pain. Most answered that they were confident they could properly prescribe opioid analgesics for CNCP (15.2% strongly agreed and 70.9% somewhat agreed). They also felt confident that they could properly treat patients with CNCP (11.8% strongly agreed and 66.0% somewhat agreed).

To the question about which opioid analgesic physicians chose as a first-line treatment in cases of mild to moderate noncancer chronic pain, 28.7% reported that they never (1.6%) or only occasionally (27.1%) prescribed one of the proposed medications. More than a third of the sample (34.5%) never (1.6%) or occasionally (32.9%) prescribed immediate-release or short-acting opioids, and 51.8% of the sample (never: 12.6%; occasionally: 39.2%) prescribed extended-release or long-acting opioids. Few participants (3.1%) reported that they never or occasionally prescribed coanalgesia. Regarding which opioids participants preferred, morphine and hydromorphone were the most popular immediate-release or short-acting opioids, followed by tramadol with acetaminophen and codeine with acetaminophen combinations (Table 3). As for extended-release or long-acting opioids, again morphine and hydromorphone were first, followed by fentanyl patches, oxycodone, and tramadol.

Of the 12 factors (Table 4) proposed that could cause participants to avoid or hesitate in prescribing opioid analgesics for CNCP, three were chosen most often by participants who reported strongly or somewhat agreeing with the statements: lack of professional support in the event of abuse or dependence (75.4%), risk of dependence (73.2%), and risk of abuse, misuse, and diversion among patients (72.5%). Many participants were also worried about risks of other cognitive and physical effects of opioids, lack of support (e.g., pain specialist, psychologist, or clinical pharmacist) for patients during pain treatment, and difficulty with making a good diagnosis.

Table 5 shows that compliance with CMQ guidelines was variable. Actions recommended in case of abuse or dependence were the least often reported with only 4.9%, 19.7%, and 20.0% of participants, respectively, answering that they often/always ask patients at risk of becoming dependent to provide urine samples to test for drugs, evaluate risks of dependence using a screening tool, and have patients at risk of becoming dependent sign treatment contracts. Rates of compliance were much higher for other CMQ recommended actions but remained suboptimal overall.

Participants were quite critical regarding their academic training, with a majority reporting that their university education related to opioid analgesics prescribing practices for CNCP was “not very” (41.7%) or “not at all” (29.2%) adequate; only 3.6% chose the category “very adequate.” In terms of university education for patient CNCP management, 44.3% reported that their training was “not very,” 29.4% “not at all,” and 2.9% “very” adequate. However, a majority had attended continuing education programs in the past five years, including 65.7% related to opioid analgesic prescription practices for patients with CNCP and 73.4% for CNCP management.

Training needs related to “patient evaluation and differential diagnoses of chronic pain syndromes” were identified as the top priority (Table 6). Nearly one-third of participants rated this topic as their first choice and almost two-thirds chose it as one of their six priorities of eight proposed topics. Three other topics were rated as first priorities by at least 10% of the sample or identified as one of the six priorities; in descending order, these topics are “evaluating and managing the risks of abuse, misuse, dependence, tolerance, and diversion,” “indications and uses of alternative medications to opioids,” and “indications and uses of various opioids.” Monitoring of patient pain was also considered an important topic, with 40.1% of participants indicating that it was one of their six choices for training needs. Training need priorities also clearly targeted special populations: seniors, patients with mental disorders, and those with a history of drug abuse.

Finally, participants' choices regarding teaching methods were diverse (Table 7). The top priority was scientific oral presentations such as conferences and lectures, with 31.9% indicating the latter method as their preferred one and nearly 60% as one of their six choices of eight possibilities. As for tools that would assist their decisions to prescribe opioid analgesics, access to a free telephone or online consultation service with a medical expert was the first choice for 41.4% of participants. Access to other online resources was selected by 41.2%, with 28.6% choosing online reference tools on Québec or Canadian medical guidelines concerning opioid use for CNCP and 12.6% choosing online directories of resources and references on CNCP management and use of opioids. Prescription monitoring programs and free telephone or online consultation services with pharmaceutical experts were the first choices of 10.2% and 6.3% of participants, respectively.

## 4. Discussion

This is the first study addressing the practices and training needs of Québec family physicians in the field of CNCP. One of the main findings relates to what appears to be a discrepancy between physicians' concerns regarding risks of patient opioid abuse and dependence and physicians' actual practices. Only a minority always/often screened their patients for those risks using a screening tool, although concerns related to complications and lack of support in pain management were the main barriers to prescribing. Furthermore, almost half (46.4%) identified training on abuse and dependence risks assessment and management as one of their top three priorities, but only 13.7% ranked it first. The high level of concern reported about the potential for abuse and dependence is consistent with many previous studies [17, 18, 21–24]. This is understandable considering that the risk is real, with reported addiction or addiction-related aberrant behavior rates ranging from 8% to 26% among patients with chronic pain [25]. However, the reason for the discrepancy between physicians' concerns and practices is not clear and merits further investigation. It should be underlined that a recent CDC review shows that accuracy of risk assessment instruments for predicting opioid abuse or misuse was inconsistent or limited and no study has evaluated the effectiveness of risk mitigation strategies [7]. This situation might undermine physicians' confidence in current guidelines.

Our study shows that compliance of family physicians practices with CMQ guidelines was generally suboptimal for detection of risks and management of opioid abuse and dependence, but also for other recommendations. Many physicians did not systematically apply most recommendations, either for evaluation or for monitoring of patients and their conditions. Several studies showed low compliance with guidelines among physicians, indicating that merely disseminating guidelines is not a guarantee of good practices [19, 26]. Moreover, significant differences exist between available guidelines, which could explain the physicians' varied responses.

Similar to other studies, the majority of participants felt at least somewhat confident that they could properly treat patients and prescribe opioid analgesics for CNCP [17, 22, 27]. Yet, they rated their academic training on pain as relatively poor. This apparent contradiction might be explained by the observed high proportion of participants who had participated in continuing education programs in the five years prior to the survey. Constantly encountering patients with chronic pain may lead physicians to compensate for their deficient university training in this area. According to the literature, pain education for North American medical students is limited, variable, and fragmented [28]. These findings call for more effort on the part of universities and certifying boards to improve physicians' knowledge and practices in the field of pain.

Studies show that many physicians consider that more education is essential to improve pain management, and they value these programs highly [19, 22]. In this study, more than two-thirds of participants had taken continuing education programs on opioid analgesic prescription practices and nearly three-quarters on chronic CNCP management. As for training needs, considering the aging population, it is reassuring to observe that physicians identified seniors as the priority group among the special populations proposed. This involves training on the specific needs of this particularly vulnerable population, especially regarding dosage, monitoring, and drug interactions. Aside from the subject of “patient evaluation and differential diagnoses of chronic pain syndromes,” picked by one-third of the sample as the number one training priority, training themes were diverse and addressed several topics mostly related to indications and uses of medications (opioids per se and alternative medications to opioids). This might reflect poor training in these areas, perceived or real, among family physicians in Québec. It might also signal certain ignorance of the alternatives to medications or the perception that these are other professionals' responsibilities. Unfortunately, despite great interest from physicians, there is still limited evidence that educational interventions alone have long-term positive impacts on prescribing practices [29]. Research into effective education strategies remains a priority and recent work investigating appropriate modalities is promising [30]. Face-to-face activities seem to be the most popular option among Québec family physicians. Identifying methods that are both appealing and efficient to reach a large number of physicians remains a challenge.

It is possible that though necessary, continuing education is not sufficient; perhaps reinforcement and supportive measures are needed. In this study, the resource considered most relevant to deciding to prescribe opioids was access to a free telephone or online consultation service with a medical expert (42%). This is consistent with Allen et al.'s study (2013) which showed that 84% of Canadian family physicians found this “enabling factor” important to improve their practices [17]. Although in that study participants did not have to choose the most relevant factor, this choice was second in terms of frequency. Implementing and broadening such consultation services remain a challenge given that experts in pain medicine are rare in Canada. Financial and legal aspects of remote expert consultation also need to be addressed to ensure these services are used and ultimately improve the care of patients with CNCP.

Only 10% of the survey participants considered prescription monitoring programs as relevant to their decisions to prescribe opioids, a sharp contrast with the figure of 87% found by Allen et al. [17]. It should be mentioned that Québec does not have a prescription monitoring program yet, which could explain these results. However, perhaps upgrading and adapting the Dossier Santé Québec (an electronic platform provided by the Ministère de la Santé et des Services Sociaux du Québec) could be done; this source already contains clinical information about prescribed medications for each patient consulting a physician in the province. Finally, free telephone or online access to clinical pharmacist consultants was the first choice of only 6% of our survey participants. Pharmacists have played an important safety role in validating and distributing POs and monitoring drug therapy of their patients for years, and this contribution should be enhanced and valued [31]. Moreover, clinical pharmacists already provide pain management consultations to inpatients and outpatients (e.g., palliative care, postoperative care) [32–34]. Improved training in detection and management of patients with addiction problems and infrastructures to facilitate their clinical practices in community settings [35, 36] are needed. Interdisciplinary work emphasizing physician-pharmacist collaboration should also be strongly encouraged [31].

A substantial number of physicians often or always prescribed POs as a first-line choice in cases of mild to moderate CNCP. Among participants who prescribed POs, morphine and hydromorphone were the most popular choices either as short- or as long-acting formulations. It should be noted that, in Québec, tramadol and buprenorphine patches are not covered by the public drug reimbursement program. Furthermore, codeine is generally not recommended because of frequent drug interactions and risks of toxicity due to CYP2D6 polymorphisms. About two-thirds of participants reported that they prescribed fentanyl patches even as a first-line medication; this is somewhat surprising, since fentanyl is generally recommended for moderate to severe pain.

This study has a number of limitations. Participants were not randomly recruited and the participation rate was only 7%, thereby limiting the generalizability of our findings. Family physicians doing clinical teaching and those prescribing methadone were overrepresented in the sample, which suggests that physicians who are more interested and have better training in the field of CNCP and prescription opioids responded. However, the sample of participants was fairly large and comparable to the population of reference with respect to age and sex distribution. Furthermore, data were collected through self-reports, which could have led to a social desirability bias. Yet, the impact of such bias was probably limited by the anonymity of the study. Due to anonymity, it was impossible to prevent or check for duplicate participation. However, there is no reason to believe that physicians completed the survey several times. More research is needed to better understand the complexity of physicians' prescribing behaviours [37]. However, the results show that Québec physicians are generally comparable to other physicians both within the country and elsewhere, at least in terms of barriers, compliance to guidelines, and training needs.

In conclusion, this study provides important insights into Québec family physicians' concerns, practices, and needs with respect to CNCP management. It also sheds light on difficulties around the application of current strategies to mitigate the problem of misuse, abuse, and addiction to opioid drugs and its consequences. The need to better educate physicians in the field of pain starting from the predoctoral level cannot be emphasized enough. Basic training in the field of addiction should also be provided to all physicians. Furthermore, it is increasingly obvious that better pain management requires an interdisciplinary approach. The contribution of other health professionals with complementary expertise, including clinical pharmacists, nurses, physiotherapists, and psychotherapists, should be encouraged.

## Figures and Tables

**Table 1:** Sex and age distribution of the Québec family physician population and sample (*N* = 636).

	Family physician population^*¥*^ (%)	Survey participants (%)
Male	46	43.0
Age		
≤29 years	6	5.8
30–39 years	20	20.0
40–49 years	19	20.0
50–59 years	29	29.4
60–69 years	21	20.4
≥70 years	5	4.5

^*¥*^Data provided by Collège des Médecins du Québec.

**Table 2:** Clinical practice profile of survey participants (*N* = 636).

	Number	%^*∗*^
Number of years in clinical practice^*¥*^		
≤4 years	97	13.85
5–9 years	73	11.3
10–14 years	44	7.4
15–19 years	62	11.04
20–24 years	71	11.3
≥25 years	288	45.2
Current location of clinical practice		
Hospital	278	43.7
Emergency department	95	14.9
Outpatient clinic	50	7.97
Inpatient clinic	213	33.4
Local community service centre (CLSC)	154	23.8
Residential and long-term care centre (CHSLD)	105	16.5
Family medicine group (FMG)	328	51.5
Private clinic	223	35.7
Group	164	26.2
Solo	60	9.7
Clinical teaching^‡^		
Yes	347	54.8
No	282	45.2
Number of patients per week^*£*^		
≤49	211	33.1
50–99	320	50.5
100–149	93	14.8
≥150	10	1.5
Has full exemption to prescribe methadone		
Yes, for pain	65	10.34
Yes, for substitution treatment	24	3.63
Yes, for both pain and substitution treatment	32	5.25
No	515	80.77

^*∗*^Weighted proportions; ^*¥*^1 missing; ^‡^7 missing; ^*£*^2 missing.

**Table 3:** Prescription opioid preferences.

	Always	Frequently	Occasionally	Never
	%^*∗*^	%^*∗*^	%^*∗*^	%^*∗*^
Short-acting drugs				
Morphine	1.2	34.7	48.9	15.3
Hydromorphone	1.25	33.2	48.9	16.7
Tramadol/acetaminophen	1.02	23.7	51.0	24.3
Codeine/acetaminophen	1.1	18.1	44.9	36.0
Oxycodone	0.7	13.7	47.4	38.3
Tramadol	0.3	14.1	44.3	41.4
Codeine	0.5	7.3	35.4	56.9
Oxycodone/acetaminophen	0.2	3.7	23.4	72.8
Hydrocodone	0.4	1.7	11.0	87.0
Tapentadol	0.2	1.1	10.2	88.5
Meperidine	0.2	0.6	9.1	90.2
Nasal butorphanol	0	0.4	1.5	98.1
Long-acting drugs				
Morphine	1.0	25.1	45.2	28.8
Hydromorphone	0.5	26.4	38.8	34.4
Transdermal fentanyl	0.3	15.5	42.1	42.0
Oxycodone	0.3	12.0	44.8	42.9
Tramadol	0.2	14.9	41.8	43.1
Codeine	0	5.1	27.6	67.3
Transdermal buprenorphine	0.2	3.0	21.8	75.1
Tapentadol	0.3	1.7	9.9	88.5
Methadone	0	2.0	5.6	92.5

^*∗*^Weighted proportions.

**Table 4:** Barriers to opioid analgesic prescription (*N* = 636).

	Strongly agree	Somewhat agree	Somewhat disagree	Strongly disagree	Does not apply
	%^*∗*^	%^*∗*^	%^*∗*^	%^*∗*^	%^*∗*^
Difficulty in properly evaluating/establishing a clear diagnosis of CNCP	13.1	41.7	32.7	10.8	1.7
Extra work linked to opioid treatment	6.34	23.2	42.9	24.9	2.3
CNCP patients tend to be “heavy cases”	13.2	34.2	31.9	18.2	2.6
Adverse effects	19.62	50.1	24.6	4.8	1.0
Risk of abuse, misuse, or diversion	26.9	45.6	22.9	3.6	1.1
Risk of dependence	29.3	43.9	20.4	5.3	1.2
Risk of overdose	11.7	35.0	41.5	10.3	1.6
Risk of tolerance	16.8	41.7	31.2	9.5	0.8
Lack of professional support in treating the pain	30.0	36.2	24.1	7.9	1.8
Lack of professional support in the event of abuse or dependence	36.1	39.3	17.2	4.8	2.7
Risk of inspection or professional sanctions	3.3	15.1	40.2	39.2	2.3
Lack of proof that opioid analgesics are effective for CNCP in the long term	8.6	28.4	41.7	18.2	3.10

^*∗*^Weighted proportions.

**Table 5:** Rates of compliance to the CMQ guidelines (*n* = 636).

	Always	Frequently	Sometimes	Never	Not applicable
	%^*∗*^	%^*∗*^	%^*∗*^	%^*∗*^	%^*∗*^
Prescribe a urine test for drug testing to patients at risk	1.0	3.9	21.6	62.0	11.5
Assess risk of dependence using a screening tool	5.11	14.6	35.7	44.5	—
Sign a contract with patients at risk	7.4	12.7	25.2	42.4	12.4
Perform a psychosocial assessment	23.6	35.8	32.2	8.5	—
Use a scale to assess the intensity of pain	28.9	37.7	26.9	6.5	—
Use a tracking sheet	52.4	17.3	15.0	15.3	—
Assess patient's overall level of function	35.3	43.4	16.6	4.7	—
Inform patients & their close contacts of the risks	46.3	36.7	13.2	3.8	—
Develop a treatment plan with follow-ups	48.9	35.8	12.8	2.5	—
Progressively reduce the dosage when the patient improves	45.4	40.2	11.9	2.4	—
Discuss additional or alternative treatments	51.6	36.6	10.7	1.2	—
Do a complete anamnesis	70.5	23.3	5.9	0.2	—

^*∗*^Weighted proportions.

**Table 6:** Participants' priorities of training needs (*N* = 636).

	Top priority %^*∗*^	One of the six priorities %^*∗*^
Topics^§^		
Indications and uses of various opioids	17.7	43.1
Indications and uses of alternative medications to opioids	14.1	45.2
Evaluating and managing the risks of abuse, misuse, dependence, tolerance, and diversion	13.7	46.4
Evaluating and managing side effects	2.5	16.8
Patient evaluation and differential diagnoses of chronic pain syndromes	31.7	60.5
Monitoring patient pain (patient reevaluation, treatment plan adjustment, etc.)	8.7	40.9
Indications and uses of nonpharmacological approaches (psychotherapy, physiotherapy, occupational therapy, etc.)	5.0	24.3
Indications and methods for referring patients to a multidisciplinary pain clinic	6.2	23.4
Other	0.4	—
Special populations^§^		
Infants and toddlers	0.2	1.6
Preschoolers	0.46	1.0
School-age children	0.1	3.3
Adolescents	0.6	10.61
Pregnant women	2.0	9.5
CNCP patients with mental health disorders	30.3	9.5
Patients with a history of drug abuse	25.9	91.4
Seniors	40.5	90.1

^*∗*^Weighted proportions; ^§^12 missing.

**Table 7:** Priorities of teaching methods (*n* = 636).

Teaching methods^§^	Top priority %^*∗*^	One of the six priorities %^*∗*^
Interactive classroom activities (problem-based learning, case study discussions, etc.)	20.0	44.7
Scientific oral presentations (conferences, lectures, etc.)	31.9	59.9
Webinars (online presentations)	6.8	32.6
Intensive courses (classroom-based)	4.1	18.4
Online courses with tutoring	5.8	19.3
Online self-study modules without tutoring	12.2	42.4
Self-study modules (on paper)	4.5	23.3
Internship in a pain management clinic	7.4	21.9
Videos on the best practices for prescribing opioid analgesics	7.2	37.7

^*∗*^Weighted proportions; ^§^5 missing.
